# *Mycobacterium tuberculosis* Infection and Tissue Factor Expression in Macrophages

**DOI:** 10.1371/journal.pone.0045700

**Published:** 2012-09-24

**Authors:** Hema Kothari, L. Vijaya Mohan Rao, Ramakrishna Vankayalapati, Usha R. Pendurthi

**Affiliations:** 1 Department of Cellular and Molecular Biology, The University of Texas Health Science Center, Tyler, Texas, United States of America; 2 Center for Pulmonary and Infectious Disease Control, The University of Texas Health Science Center, Tyler, Texas, United States of America; Tulane University, United States of America

## Abstract

A number of earlier studies reported the occurrence of thrombotic complications, particularly disseminated intravascular coagulation and deep vein thrombosis, in tuberculosis (TB) patients. The aberrant expression of tissue factor (TF), the primary activator of coagulation cascade, is known to be responsible for thrombotic disorders in many diseases including bacterial infections. Further, expression of TF by cells of the monocyte/macrophage lineage is also shown to contribute to the development and progression of local and systemic inflammatory reactions. In the present study, we have investigated whether *Mycobacterium tuberculosis (Mtb)* infection induces TF expression in macrophages, and various host and pathogenic factors responsible for TF expression. We have tested the effect of live virulent *Mtb* H37Rv, gamma-irradiated *Mtb* H37Rv (γ*-Mtb*) and various components derived from *Mtb* H37Rv on TF expression in macrophages. The data presented in the manuscript show that both live virulent *Mtb* and γ*-Mtb* treatments markedly increased TF activity in macrophages, predominantly in the CD14^+^ macrophages. Detailed studies using γ*-Mtb* showed that the increased TF activity in macrophages following *Mtb* treatment is the result of TF transcriptional activation. The signaling pathways of TF induction by *Mtb* appears to be distinct from that of LPS-induced TF expression. *Mtb*-mediated TF expression is dependent on cooperation of CD14/TLR2/TLR4 and probably yet another unknown receptor/cofactor. *Mtb* cell wall core components, mycolyl arabinogalactan peptidoglycan (mAGP), phosphatidylinositol mannoside-6 (PIM6) and lipomannan (LM) were identified as factors responsible for induction of TF in the order of mAGP>PIM6>LM. A direct contact between bacteria and macrophage and not *Mtb-*released soluble factors is critical for TF induction by *Mtb*. In summary, our data show that *Mtb* induces TF expression in macrophages and *Mtb* signaling pathways that elicit TF induction require cooperation of multiple receptors, co-receptors/co-factors including Toll-like receptors. The importance of TF in granuloma formation and containment of *Mtb* is discussed.

## Introduction

Activation of extrinsic coagulation cascade initiated by tissue factor (TF) is a critical step in the pathogenesis of various thrombotic disorders [1], [2]. Under resting conditions, cells that come in direct contact with blood such as endothelial cells and monocytes do not express TF [3], [4] but a variety of pathological stimuli, particularly bacterial infections, may induce TF expression in these cells [5], [6]. The aberrant expression of TF by cells of the monocyte/macrophage lineage is a major contributor to the development and progression of local and systemic inflammatory reactions in many diseases, including sepsis [7]–[9], endotoxemia [10]–[12], active coronary heart disease [13], [14], and atherosclerosis [15]. Blockade of TF activity was shown to decrease procoagulant response, pulmonary fibrin deposition, and cytokine expression in various models of bacterial-induced lung inflammation [16]–[19]. Tissue factor, in addition to activating the coagulation cascade, can also influence many other cellular functions by supporting FVIIa and downstream protease induced cell signaling *via* activation of protease-activated receptors (PARs) [20]–[22].

Tuberculosis (TB), a disease caused by *Mycobacterium tuberculosis (Mtb)* infection, affects nearly one third of the world's population [23]. In addition, TB is a leading killer of immune compromised people such as those infected with HIV [24]–[26]. A number of studies have reported the occurrence of thrombotic complications in TB patients, particularly disseminated intravascular coagulation (DIC) and deep vein thrombosis (DVT) [27]–[32]. However, it is unclear how tuberculosis infection causes thrombotic complications in some patients as mycobacteria are not known to produce endotoxins or exotoxins that are known to initiate the clotting cascade. Although limited number of studies in the past have shown that *in vitro* infection of monocytes with mycobacterial components can induce production of the proinflammatory cytokines and increase the procoagulant activity [33], [34], there is little information on the regulatory pathways and molecular mechanisms responsible for increased TF expression during mycobacterial infections. Earlier studies have reported that cell wall components of *Mycobacterium* species induced TF expression in macrophages, but these studies were limited to the use of derivatives from non-virulent *Mycobacterium* species. [33], [35], [36]. Further, Moller et al. [35] had reported that non-mannose-capped lipoarabinomannan (AraLAM) from rapidly growing non-pathogenic *Mycobacterium* species but not mannose-capped lipoarabinomannan (ManLAM) from virulent H37Rv *Mtb* strain induced TF and TNF-α expression in human peripheral monocytes. We are not aware of any studies that examined expression of TF in macrophages in response to live virulent *Mtb* and identified the cell wall component(s) of *Mtb* that are responsible for TF induction in macrophages or macrophage receptors that mediate *Mtb* induction of TF.

The present study was undertaken to evaluate the effect of *Mtb* infection in inducing TF procoagulant activity in human macrophages and to delineate the molecular basis of *Mtb-*induced TF expression in macrophages. Furthermore, the relation between bacterial virulence and procoagulant potential was investigated by comparing the abilities of virulent H37Rv and avirulent H37Ra to induce TF expression. Results of our studies showed that exposure of human monocyte-derived macrophages (MDMs) to live *Mtb* or gamma-irradiated *Mtb* H37Rv (γ*-Mtb*) led to a marked induction of TF in MDMs, predominantly in the CD14^+^ macrophage subpopulation. The kinetics and mechanism of *Mtb-*induced TF expression differs from that of lipopolysaccharide (LPS)-induced TF expression in macrophages. The data presented in the manuscript indicate that *Mtb* signaling pathways that elicit TF induction require cooperation between multiple receptors, co-receptors/co-factors including Toll-like receptors.

## Results

### *γ-Mtb* increased TF activity predominantly in CD14^+^ macrophages and is much more potent than bacterial LPS

**Figure 1:**
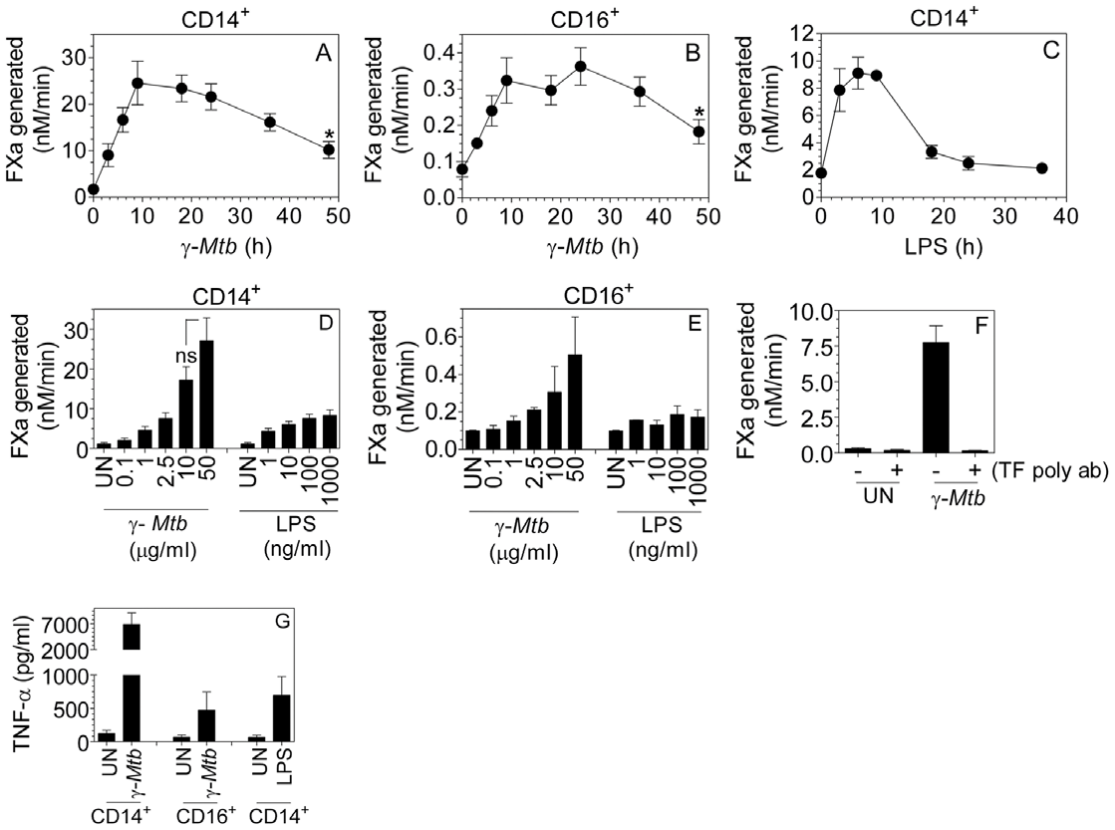
γ-irradiated H37Rv *Mtb* (*γ-Mtb*) induces TF expression in macrophages. MDMs were stimulated for varying time periods with γ-*Mtb* H37Rv (10 µg/ml, **A** and **B**) or LPS (100 ng/ml, **C**) in complete RPMI medium. TF activity in the cell lysates was measured in factor X activation assay. **Please note differences in Y-axis scale in three panels.** CD14^+^ (**D**) CD16^+^ (**E**) MDMs were treated with varying doses of γ-*Mtb* H37Rv or LPS for 9 h or 6 h, respectively, and TF activity in the cell lysates was determined in factor X activation assay. (**F**) TF activity in the cell lysates of CD14^+^ treated with γ-*Mtb* for 9 h was measured in the presence or absence of anti-TF polyclonal antibodies (10 µg/ml) (**G**) Cell supernatants of MDMs treated with γ-*Mtb* (10 µg/ml) for 9 h or LPS (100 ng/ml) for 6 h were used to measure TNF-α using ELISA. * denotes significant difference from untreated control for all time points in both CD14^+^ and CD16^+^ (P<0.05). Data are mean ± SEM (n = 3–6).

CD14^+^ and CD16^+^ MDMs were treated for varying time periods with γ-*Mtb* (10 µg/ml) or LPS (100 ng/ml) and TF expression in MDMs was evaluated by measuring TF procoagulant activity in the cell lysates. As shown in Fig. 1A and 1B, γ-*Mtb* treatment increased TF activity in both CD14^+^ and CD16^+^ MDMs. Although the time course for TF activity increase was similar in both CD14^+^ and CD16^+^ macrophages, the level of induction was 100 times higher in CD14^+^ MDMs in comparison to that observed in CD16^+^ macrophages (Fig. 1B). Upon γ-*Mtb* treatment, TF activity increased gradually in MDMs, reaching the maximal at about 10 h. Although the increased TF activity in γ-*Mtb*-treated cells was reduced slowly thereafter, substantial amount of the increased TF activity was retained for a prolonged time period. At the end of 48 h, TF activity in γ-*Mtb*-treated MDMs was about 5-fold higher than that of the unperturbed MDMs. This differs from the kinetics of LPS-induced TF activity, which reached peak at about 4 to 6 h and then declined close to the basal levels by 18 h (Fig. 1C). The increase in TF activity in MDMs was dose-dependent in both γ-*Mtb* and LPS-treated MDMs (Fig. 1D and 1E). At optimal or close to optimal concentrations, γ-*Mtb* treatment increased TF activity in MDMs 3- to 4-fold higher than that obtained with LPS treatment. Incubation of γ-*Mtb-*treated MDMs with anti-TF antibodies completely abrogated FX activation, indicating that the increased procoagulant activity measured in γ-*Mtb-*treated MDMs comes entirely from increased TF expression (Fig. 1F). Similar to TF, TNF-α production was also much higher in CD14^+^ macrophages as compared to CD16^+^ and γ-*Mtb* stimulation led to a more pronounced TNF-α secretion than LPS (Fig. 1G). Since CD16^+^ macrophages are a minor population that attach to the cell culture plates and did not show much induction of TF in comparison to CD14^+^ macrophages, further experiments were conducted with only MDMs that were prepared by the plate adherence method.

### *γ-Mtb* treatment changed the non-coagulant phenotype of endothelial cells to procoagulant

**Figure 2:**
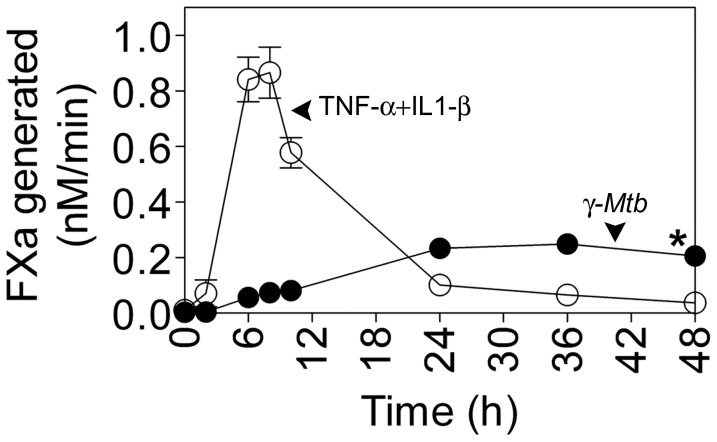
*γ-Mtb* induces TF expression in endothelial cells which follows different kinetics than TNF-α+IL1-β. HUVEC were stimulated with TNF-α+IL1-β (20 ng/ml each) (open circles) or *γ*-*Mtb* H37Rv (10 µg/ml) (closed circles) in EBM-2 complete medium for varying time periods. At the end of the treatment, TF activity on intact monolayers was measured in factor X activation assay. * denotes significant difference from untreated control for all time points (P<0.001). Data are mean ± SEM (n = 3).

We also evaluated the capacity of *γ-Mtb* to induce TF expression in endothelial cells. HUVEC were treated with *γ-Mtb* or TNF-α+IL1-β (as a positive control) for varying time periods. As shown in Fig. 2, *γ-Mtb* significantly induced TF activity at 6 h which increased gradually reaching maximum by 24 h (20-fold higher over the basal value) and was maintained at significantly constant higher levels over basal values up to 48 h. Although TNF-α+IL1-β-mediated TF activity response was faster and 3.5 times higher than that induced by *γ-Mtb*, it declined rapidly after 8 h reaching basal levels by 24 h or later time periods.

### *De novo* synthesis of TF mRNA and protein in MDMs treated with *γ-Mtb*

**Figure 3:**
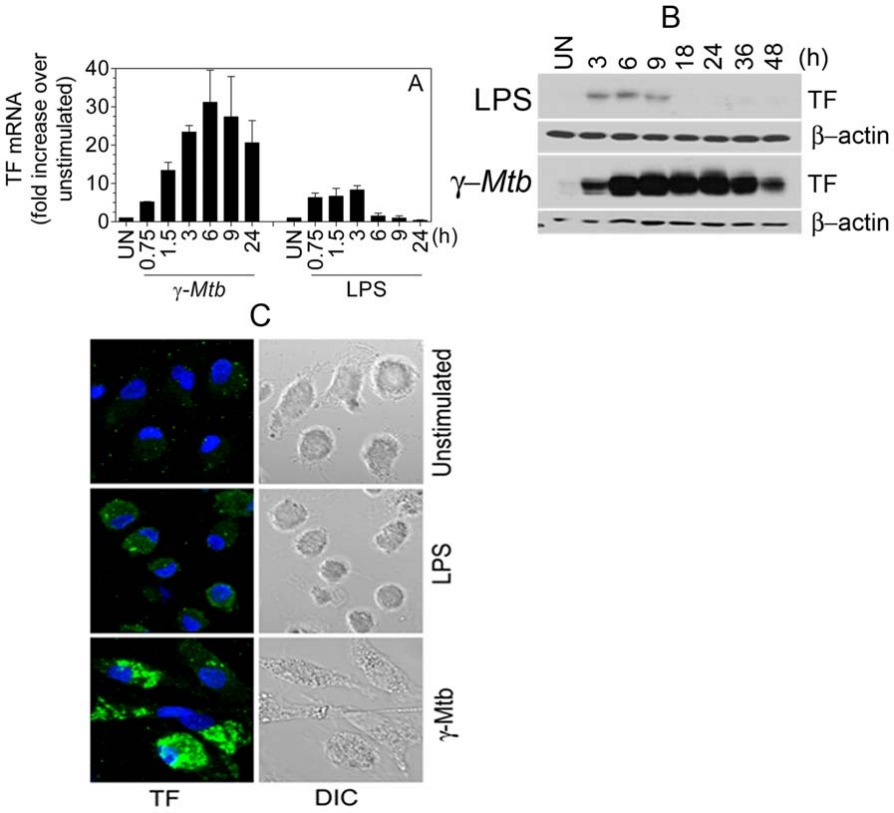
Expression of TF mRNA and protein synthesis in macrophages in response to treatment with *γ-Mtb*. (**A**) MDMs in 6-well plates were stimulated with γ-*Mtb* H37Rv (10 µg/ml) or LPS (100 ng/ml) for varying time periods in RPMI complete medium. Total RNA was isolated and subjected to real-time qPCR analysis in triplicates. GAPDH was used as an internal control and data were normalized to the internal control. TF mRNA increase was depicted as fold increase over unstimulated cells (**B**) Immunoblotting of MDM cell lysates harvested following γ-*Mtb* H37Rv (10 µg/ml) or LPS (100 ng/ml) treatment for varying times with TF9C3 mAb. (**C**) Immunofluorescence confocal microscopy with TF polyclonal antibodies (10 µg/ml) showing increased macrophage TF expression upon γ-*Mtb* stimulation as compared to control and LPS stimulation. Data are mean ± SEM (n = 3) or a representative picture.

To examine whether the increased TF activity in MDMs in response to *γ-Mtb* treatment was a result of *de novo* synthesis of TF, *γ-Mtb-*stimulated macrophages were evaluated for TF expression both at the transcription and translation levels. As shown in Fig. 3A, both γ-*Mtb* and LPS effectively upregulated TF mRNA expression but with different kinetics. Exposure of macrophages to γ-*Mtb* caused a 5 fold induction of the TF transcript at 45 min which markedly increased with increasing time points reaching ∼30 fold at 6 h; increased TF mRNA levels were sustained for more than 24 h. In contrast, rapid and transient induction of TF mRNA was seen in LPS-treated MDMs, reaching maximal of 5–8-fold over the basal level at 45 min and returned to baseline by 6 h (Fig. 3A). In agreement with mRNA expression, TF antigen expression was also much higher in γ-*Mtb* stimulated macrophages as compared to LPS-treated macrophages, and the protein was stable up to 24 h where as TF protein started to decline by 9 h in LPS-stimulated macrophages and reached to basal/undetectable levels by 18 h (Fig. 3B). Immunofluorescence confocal microscopy also showed much intense staining of TF in γ-*Mtb-*stimulated macrophages as compared to LPS-stimulated macrophages (Fig. 3C).

### *γ-Mtb*-mediated TF induction and TNF-α secretion is mostly independent of CD14

**Figure 4:**
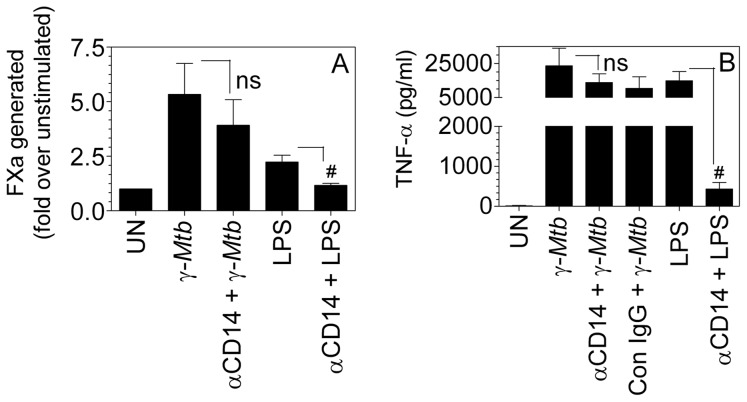
*γ-Mtb*-mediated TF induction and TNF-α secretion is mostly independent of CD14. MDMs were either treated with γ-*Mtb* H37Rv (10 µg/ml) for 9 h or LPS (100 ng/ml) for 6 h in the presence or absence of CD14 blocking antibodies (10 µg/ml) (where CD14 antibody was added, the cells were preincubated with it for 45 min before stimulation). At the end of stimulation period, cell free supernatants were collected for TNF-α ELISA and the cells were used to measure cell surface TF activity. (**A**) TF activity as measured in FXa generation. Data is depicted as fold increase in TF activity over control (**B**) TNF-α levels as determined in ELISA. ns, not statistically significant; # indicates statistically significant decrease compared to LPS alone (*P*<0.05). Data are mean ± SEM (n = 3–6).

As mentioned above, of the two monocyte subpopulations, the one expressing predominantly CD14 receptor on the cell surface showed marked TF induction whereas the CD16^+^ cells expressing low levels of the CD14 receptor, though susceptible for TF expression, showed weak induction, indicating that γ-*Mtb*-mediated TF induction involves the activation of CD14 receptor pathway. To ascertain this, macrophages were first incubated with CD14 neutralizing antibodies for 1 h and then stimulated with γ-*Mtb* for 9 h or LPS for 6 h (used as a positive control). As shown in Fig. 4A, LPS-mediated TF induction was completely abrogated by CD14 antibodies. In contrast to LPS induction, blockade of CD14 reduced *Mtb-*mediated TF induction only modestly, which was statistically insignificant. Similar to the TF activity data, blocking CD14 in LPS-treated cells resulted in a marked reduction of the TNF-α secretion where as there was only an insignificant decrease in the TNF-α levels in γ-*Mtb* stimulated macrophages (Fig. 4B).

### The role of toll-like receptors in *γ-Mtb* -induced TF expression in macrophages

**Figure 5:**
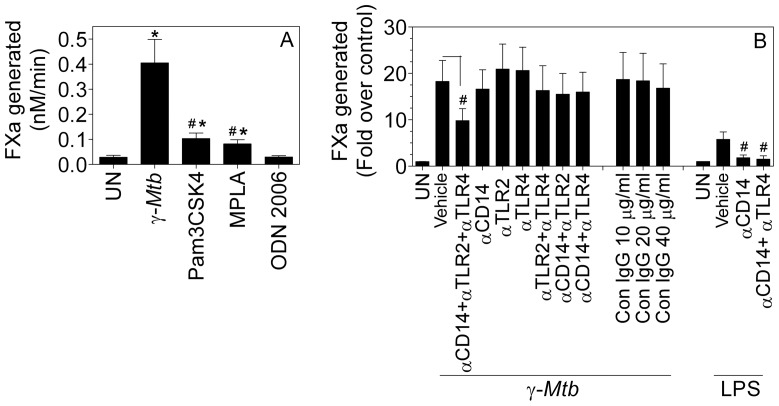
TLR2 and TLR4 along with CD14 receptor are partly responsible for *γ-Mtb*-mediated TF induction. (**A**) MDMs were treated with TLR2 agonist (Pam3CSK4, 1 µg/ml), TLR4 agonist (MPLA, 1 µg/ml) and TLR9 agonist (ODN2006, 10 µg/ml) for 9 h. As a control, MDMs were also treated with γ-*Mtb* H37Rv (10 µg/ml). Cell surface TF activity was determined in factor X activation assay.* indicates significant difference compared to untreated cells (P≤0.01); # denotes significant difference compared to γ-*Mtb* treated cells (P<0.01) (**B**) MDMs were preincubated with CD14, TLR2 and TLR4 blocking antibodies (each, 10 µg/ml) either alone or in combination and with isotype control IgG for 45 min at 37°C and then cells were stimulated with γ-*Mtb* for 9 h. At the end of the stimulation period cell surface TF activity was measured in factor X activation assay. # denotes significant inhibition compared to γ-*Mtb* or LPS alone treated cells (P<0.05); paired t-test. Data are mean ± SEM (n = 3–8).

Although CD14 inhibition had only a modest influence on *γ-Mtb-*mediated TF expression, it may still have a role, in conjunction with TLRs in inducing TF. To determine the potential role of TLRs in mediating *Mtb*-induced TF expression in macrophages, first we have investigated the effect of TLR activation on TF induction in macrophages. MDMs were stimulated with TLR2 (Pam3CSK4, 1 µg/ml), TLR4 (Mono-phosphoryl lipid A, MPLA, 1 µg/ml) or TLR9 (ODN 2006, 5 µM) agonists for 9 h in parallel with γ-*Mtb* (10 µg/ml). As shown in Fig. 5A, both TLR2 and TLR4 agonists led to a significant 2 to 3-fold induction of TF activity whereas TLR9 agonist had no significant effect on TF expression. In comparison, in parallel experiments γ-*Mtb* treatment increased TF activity in MDMs by about 15-fold.

Next, to determine the potential role of TLR2 and TLR4 alone or in combination with CD14 in mediating γ-*Mtb* induction of TF, MDMs were treated with function neutralizing antibodies to TLR2 and TLR4 alone, together or in combination with CD14 antibodies before they were exposed to γ-*Mtb*. As shown in Fig. 5B, there was no inhibition of γ-*Mtb-*mediated TF induction by blockade of TLR2 or TLR4 alone; and slight and statistically insignificant inhibition (∼15%) by blockade of both TLR2 and TLR4. Inhibition of all three receptors - TLR2, TLR4 and CD14 simultaneously with neutralizing antibodies significantly reduced γ-*Mtb-*induced TF activity by 50%. In positive control experiments, CD14 antibodies alone or together with TLR4 antibodies completely blocked LPS-mediated TF induction. Overall these data indicate that γ-*Mtb*-mediated TF induction is dependent on complex interactions of some yet unidentified receptor with TLRs and CD14. Alternatively, γ-*Mtb*-induced marked increase in TF activity may be a result of cumulative effect of multiple and independent pathways, including CD14 and TLR pathways.

### Proinflammatory cytokines produced upon mycobacterial infection do not play a role in induction of TF in macrophages

**Figure 6:**
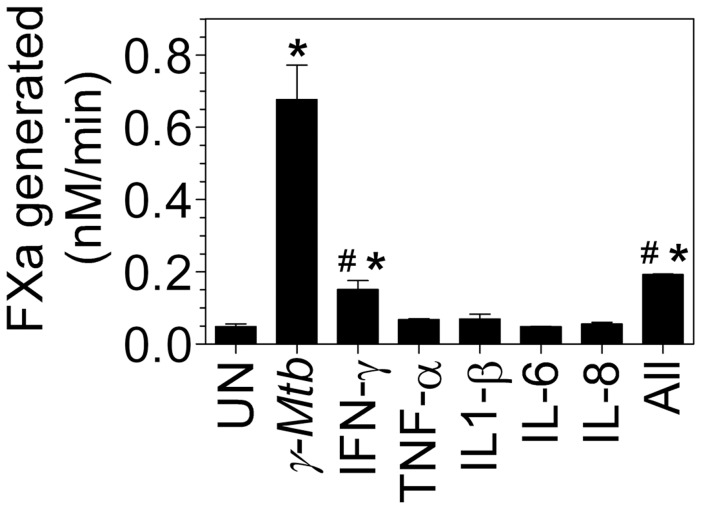
Effect of various cytokines, in comparison with *γ-Mtb*, on induction of TF in macrophages. MDMs were treated with IFN-γ (100 ng/ml), TNF-α (20 ng/ml), IL1-β (20 ng/ml), IL-6 (20 ng/ml) or IL-8 (20 ng/ml) for 9 h. In parallel, MDMs were treated with γ-*Mtb* (10 µg/ml). Cell surface TF activity was determined in factor X activation assay. * indicates significant difference compared to untreated control (P<0.05); # denotes significant difference compared to γ-*Mtb* treated cells (P<0.05). Data are mean ± SEM (n = 3).

Mycobacterial infection results in macrophage elaboration of various proinflammatory cytokines such as TNF-α, IL1-β, IL-6, IL-8 and IFN-γ [37]–[39]. Therefore, we next investigated whether these proinflammatory cytokines secreted upon macrophage stimulation with γ-*Mtb* could be responsible for TF induction. MDMs were treated exogenously with TNF-α, IL1-β, IL-6, IL-8 or IFN-γ alone or altogether and the induction of TF activity was evaluated. As shown in Fig. 6, TNF-α, IL1-β, IL-6 and IL-8 failed to induce TF expression in MDMs. IFN-γ treatment led to a significant 3-fold increase in TF activity in MDMs but it was less than 20% of that was obtained with γ-*Mtb* treatment. There was no further increase in TF induction when MDMs were treated with a combination of all these cytokines. In additional studies, to determine IFN-γ's contribution in *γ-Mtb*-mediated TF activity induction, MDMs were stimulated with γ-*Mtb* in the presence of IFN-γ neutralizing antibodies, which showed no inhibition of TF activity (data not shown).

### Effect of various mycobacterial cell components on TF induction

**Figure 7:**
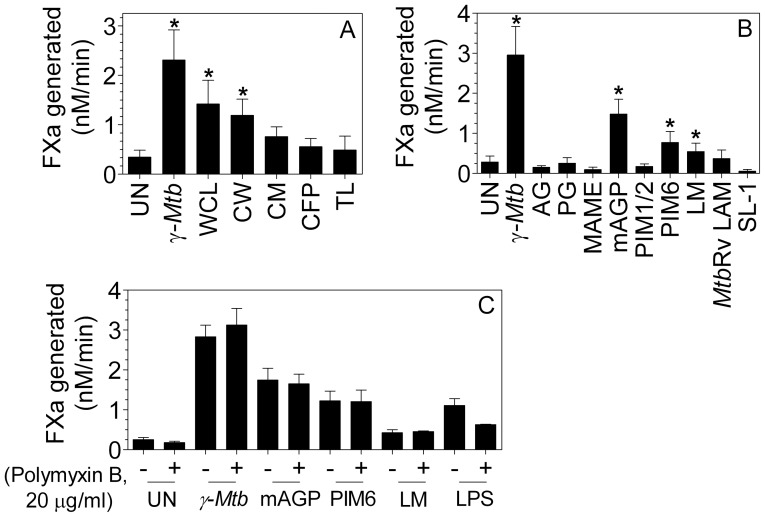
Mycobacterial mAGP, PIM6 and Lipomannans are the potential players in inducing TF in macrophages. (**A**) MDMs were treated with various mycobacterial subcellular fractions, whole cell lysate (WCL), cell wall (CW), cell membrane (CM), culture filtrate protein (CFP) and total lipids (TL), all at 50 µg/ml concentration for 9 h. * denotes significant difference compared to untreated control cells (P<0.05) (**B**) MDMs were treated with various mycobacterial purified components such as arabinogalactan (AG), peptidoglycan (PG), mycolic acid methyl esters (MAME), mycolyl arabinogalactan peptidoglycan complex (mAGP), phosphatidylionositide mannoside 1,2 and 6 (PIM1/2 and PIM6), lipomannan (LM) and lipoarabinomannan from H37Rv (LAM Rv) and sulfolipid (SL-1), all at 50 µg/ml concentration for 9 h. * denotes significant difference compared to untreated control cells (P<0.05); paired t-test (**C**) MDMs were treated with mAGP, PIM6, LM in presence and absence of polymyxin B (10 µg/ml). As a positive control for polymyxin B effect in neutralizing endotoxin, MDMs were treated with LPS (100 ng/ml) ± polymyxin B. Cell surface TF activity was determined in factor X activation assay. Data are mean ± SEM (n = 4–6).

We first investigated the ability of various mycobacterium sub-cellular fractions to induce TF expression. As illustrated in Fig. 7A, all subcellular fractions tested, including, whole cell lysate (WCL), cell wall (CW), cell membrane (CM), culture filtrate proteins (CFP) and total lipids (TL) induced TF activity to varied levels of which, former three seemed more potent as compared to the latter two.

Further, to identify the specific cell wall component, MDMs were stimulated with LAMs derived from virulent H37Rv, mAGP complex as well as purified cell wall components isolated from mAGP complex (i.e., mycolic acids, arabinogalactan, and peptidoglycan) or lipids (i.e., PIM1, PIM2, PIM6, sulfolipids). Of all the purified components tested the mycobacterial cell wall core component mycolyl arabinogalactan peptidoglycan (mAGP), phosphatidylinositol mannoside-6 (PIM6) and lipomannan (LM) showed induction of TF protein and activity in the order of mAGP>PIM6>LM (Fig. 7B). In order to confirm that the induction of TF activity by these components is not a resultant of LPS contamination, these components were preincubated with polymyxin B (10 µg/ml) for 1 h before they were added to the macrophages. As evident in Fig. 7C, polymyxin B did not diminish the ability of these components to induce TF expression. In a control experiment, polymyxin B reduced LPS-mediated TF induction.

### *γ-Mtb-*mediated induction of TF in macrophages is partly dependent on ERK and PKC pathways

**Figure 8:**
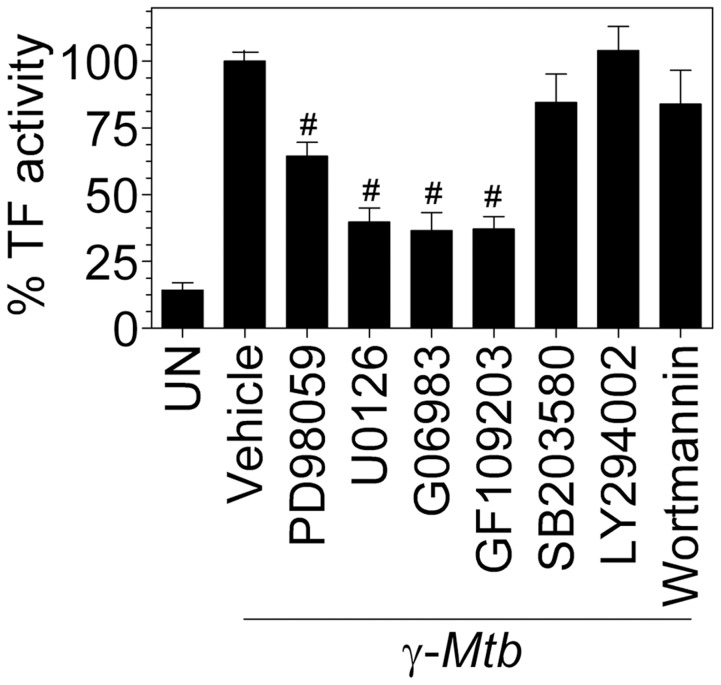
ERK and PKC pathways play a role in *Mtb*-induction of TF procoagulant activity. MDMs were pretreated with the MEK inhibitors, PD98059 and U0126 and PKC inhibitors, GF109203 and Gö6983, p38 MAPK inhibitor, SB203580 and PI3-AKT inhibitors, LY294002 and wortmannin (all used at 10 µM concentration) for 1 h. Cells were then stimulated with γ-*Mtb* (10 µg/ml) for 9 h. Cell surface TF activity measured in factor X activation assay. Values obtained for γ-*Mtb* alone treated cells are represented as 100%. # indicates significant inhibition compared to γ-*Mtb* alone treated cells (P = 0.0001). Data are mean ± SEM ( n = 7).

Signaling through the different MAP kinase families is known to regulate the expression of TF in response to numerous stimuli which differ according to the inducing agent and the cell type [14]. However, we are not aware of any previous report that examined the signaling events leading to TF upregulation in macrophages upon *Mtb* infection. To identify specific signaling pathway of *Mtb*-mediated induction of TF expression, the roles of extracellular signal-regulated kinase (ERK), p38 mitogen-activated protein kinase (MAPK), phosphoionositide 3-kinase-Akt (PI3-Akt) and protein kinase C (PKC) were investigated using following specific inhibitors, U0126 and PD98059 (for ERK), SB203580 (for MAPK), LY294002 and wortmannin (for PI3-Akt pathway), and Go6983 and GF109203 (for PKC). As shown in Fig. 8, pretreatment of cells with ERK and PKC inhibitors resulted in partial suppression of TF activity while the MAPK and PI3-Akt inhibitors failed to attenuate TF activity.

### Both virulent (H37Rv) and avirulent (H37Ra) mycobacterium tuberculosis strains induced TF activity in macrophages

**Figure 9:**
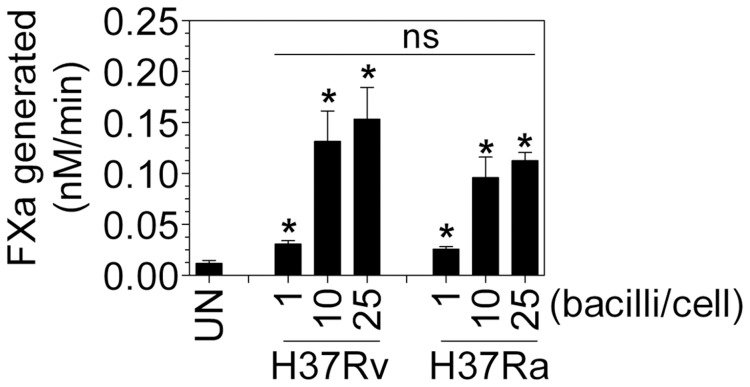
Both H37Ra and H37Rv induce TF on macrophages. MDMs in complete medium without antibiotic were infected with live H37Rv and H37Ra at varying ratios of bacteria∶cell. After 2 h, the cells were washed twice with warm RPMI serum free medium and then incubated overnight in fresh complete medium. At the end of incubation, TF activity at the cell surface was measured in factor X activation assay. ns, not statistically significant. Data are mean ± SEM ( n = 5).

To test whether *Mtb* virulence factor plays a role in TF induction, we compared the relative ability of the virulent and avirulent strains of *Mtb* for the induction of TF activity in macrophages. Macrophages were infected with 1–25 bacilli/cell using either H37Rv or H37Ra. As shown in Fig. 9, macrophages infected with increasing number of bacteria/cell of live H37Rv or H37Ra showed dose-dependent induction of TF activity. The avirulent strain, H37Ra caused 20–30% lower TF activity induction within each experiment compared to the virulent H37Rv but the difference was statistically non-significant.

### Non-engagement of autocrine signaling for TF induction

**Figure 10:**
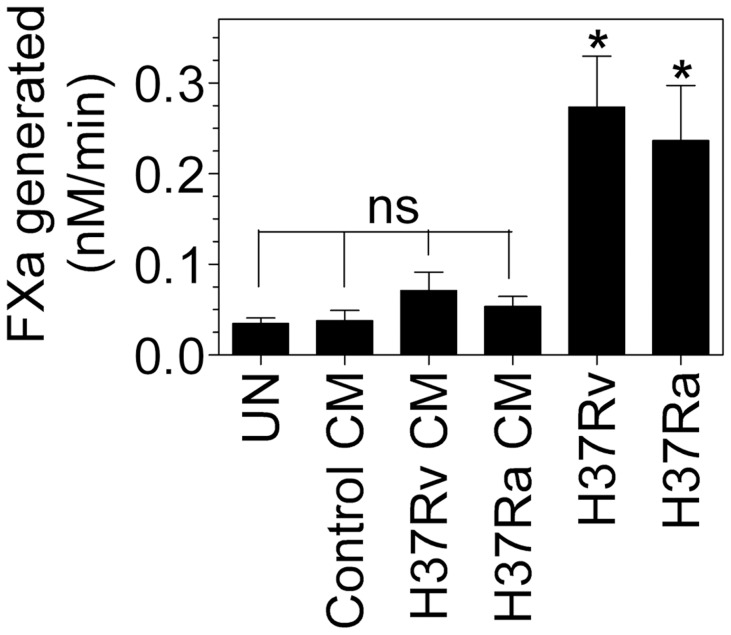
Autocrine signaling does not play a role for *Mtb*-mediated TF induction in macrophages. MDMs in 12-well plate were first infected with H37Rv or H37Ra (10 bacteria∶1 cell ratio) in 1 ml RPMI complete medium without antibiotic. After overnight, conditioned medium (CM) was collected and passed through 0.22 µm filter. MDMs in 48-well plate derived from the same donor were then incubated with 0.25 ml of the conditioned medium or live bacteria (10 bacteria∶1 cell ratio) in parallel for overnight. Thereafter, expression of TF activity at the cell surface was measured in factor X activation assay. ns, not statistically significant; * indicates significant difference compared to untreated control (P<0.005). Data are mean ± SEM ( n = 3–5).

Finally, we sought to determine whether the direct contact of the bacteria is important for TF induction or whether factors released into the medium by macrophages upon mycobacterial infection could also modulate TF expression. To answer this, macrophages were first infected with live H37Rv and H37Ra (10 bacteria/cell) for overnight and then the cell conditioned medium from uninfected or infected macrophages were collected and passed through 0.22 µm filter to remove cell debris and bacteria. This medium was then added to untreated macrophages derived from the same donor for 24 hours to assess its ability to induce TF activity. As shown in Fig. 10, conditioned medium obtained from macrophages infected with H37Rv, H37Ra or bacteria alone did not increase TF activity. Data from this and previous experiment using exogenously added cytokines showed that neither macrophage factors released into the medium (cytokines and other factors) nor soluble products of *Mtb* are capable of inducing TF expression in macrophages.

## Discussion

As a first defensive step towards any infection is the inflammatory response hoisted by the host to restrict pathogen invasion and disease spread. But in turn, the pathogen exploits and modulates the host defense mechanisms to sustain its spread and survival within the host. Activation of coagulation and fibrinolytic pathways in response to various bacterial infections are critical components elicited by both the above processes [40]–[42]. Bacterial endotoxin LPS, immune complexes and many other factors elaborated in various infectious diseases are shown to induce procoagulant TF expression in monocytes/macrophages and the endothelium, which under normal healthy state do not express TF [6], [16], [43], [44]. Cytokine and TF expression in blood circulation in response to microbial infection is shown to contribute to septic shock, intravascular coagulation, pulmonary fibrin deposition and multi organ failure in various animal models of bacterial infection [16], [18], [22], [45]. *Mycobacterium tuberculosis* is not a gram negative bacteria but is more related to the *Nocardia and Corynebacterium* species with shorter chain fatty acids. *Mycobacteria* possess a characteristic hydrophobic and waxy cell wall, rich in mycolic acids, peptidogylcan and arabinogalactan. Thus, it is quite probable that *Mycobacteria* may utilize different macrophage receptors and induce distinct signaling pathways compared to other gram positive and negative organisms.

In the present study, we show that macrophages in response to *Mtb* infection express massive amounts of TF, in quantities much higher than the known potent stimulus, the gram-negative bacterial lipopolysaccharides. However, comparing a whole organism *Mtb* preparation to LPS may not be a true comparison as the higher level of TF induction in macrophages treated with γ-irradiated *Mtb* could be a cumulative response to multiple factors in *Mtb*. The increased TF activity in macrophages in response to *Mtb* stems from *de novo* synthesis of TF. The kinetics of TF mRNA and protein expression patterns and levels differ between *Mtb* and LPS induction, with *Mtb* showing somewhat delayed but robust expression that persisted for longer time periods. Although the precise molecular mechanisms and effector signaling molecules that regulate *Mtb*-mediated transcription and translation of TF have not been resolved, herein, we have identified that *Mtb-*induced TF expression in macrophages is mediated, at least in part, by TLR2, TLR4 and CD14. In contrast to LPS stimulation, where CD14 inhibition completely abrogated TF induction as well as TNF-α secretion, CD14 inhibition only minimally reduced *Mtb-*mediated TF induction. Blockade of TLR2 and TLR4 did not prevent TF induction by *Mtb*. However, inhibition of TLR2 and TLR4 along with CD14 inhibited *Mtb-*induced TF activity by about 50%. A partial and incomplete inhibition of *Mtb-*induced TF expression by blockade of all three receptors -CD14, TLR2, and TLR4 indicate that other co-receptors/co-factors present on the surface of macrophages must cooperate with CD14/TLR2/TLR4 for *Mtb-*mediated TF induction.

Macrophages are known to synthesize and secrete cytokines, including TNF-α, IL1-β, IL-8, IL-6, IFN-γ and others, upon infection with *Mtb* [37]–[39]. Among these, TNF-α, and IL1-β are known potent inducers of TF expression in endothelial cells [46], [47]. It had been reported that TNF-α, IL1-β and IFN-γ could also induce TF expression in macrophages, but to a lesser extent [48]–[50]. Therefore, it is conceivable that TNF-α, IL1-β or IFN-γ secreted upon mycobacterial infection could be responsible for *Mtb-*mediated TF induction. However, this seems unlikely as exogenous addition of recombinant TNF-α, IL1-β, IL-6 or IL-8 failed to induce TF expression in isolated macrophages in our experiments. Although IFN-γ treatment slightly increased TF activity in macrophages, the magnitude of *Mtb-*induced TF expression far exceeds the effect of IFN-γ. Moreover, IFN-γ neutralizing antibodies failed to inhibit *Mtb-*induced TF expression. Furthermore, conditioned media from macrophages infected with either H37Rv or H37Ra that contain macrophage secreted cytokines and probably some bacteria-derived soluble factors also failed to induce TF activity when added to fresh macrophages.

Earlier studies showed that mycobacterial components can induce TF [33]–[35]. Trehalose 6, 6-dimycolate also known as cord factor is an important component modulating various macrophage functions and has been shown to induce macrophage TF and TNF-α induction [34], [51]. However, a complete analysis of other components from virulent *Mtb* responsible for TF induction is unknown. In this study, we have successfully identified the various cell wall components involved in TF induction. We found that the fraction containing only cell wall components of *Mtb can be* as potent as the *Mtb* whole cell lysate. Further, culture filtrate proteins of *Mtb* were not efficient inducers of TF expression in macrophages. These data suggest the likelihood of non-involvement of *Mtb* secreted antigens in inducing TF expression. Among the purified cell wall components analyzed, mAGP complex induced the highest levels of TF activity expression but it is interesting to note that the individual components that comprise the core mAGP such as mycolic acid, arabinogalactan and peptidoglycan individually did not show TF induction indicating towards a specific conformational requirement of the mAGP complex as a pathogen-associated-molecular-pattern (PAMP) for recognition by pattern recognition receptors on macrophages such as TLRs to induce TF expression.

Considering the fact that the two mycobacterial strains of *Mtb*, H37Ra and H37Rv differ at the genetic level, in macrophage activation mechanisms, cytokine elaboration and display completely distinct disease pathology [52]–[56], we expected that they may show differential potential for TF induction. However we found only minimal differences between these two strains in their ability to induce TF expression. It is possible that both the strains may have relatively similar composition but may have varied abundance or *vice-versa* of some of the outer cell wall components that are responsible for TF induction.

Coagulation and inflammation share a bidirectional relation playing crucial roles in host defense [9], [57]. Proinflammatory cytokines produced in infections can induce TF expression on monocytes and endothelial cells [46]–[49], [58]. In turn, TF, in addition to activating coagulation, can also amplify the production and release of inflammatory mediators by signaling mediated by TF-FVIIa complex formation and downstream proteases generated by TF-FVIIa-induced coagulation [59]–[62]. Ample studies conducted have provided strong evidences that by blocking TF, the proinflammtory cytokine elaboration (IL-6, IL-8), levels of soluble TNF receptor-1 and infection-associated organ injury and mortality were reduced [63]–[65]. It will be interesting to examine in future whether *Mtb*-induced TF plays a role in propagation of inflammatory responses induced by *Mtb*.

Most of the studies that investigated the potential role of TF in modulating inflammation and *vice-versa* were limited to acute infection and inflammatory settings [9], [66]–[68]. Our present study indicates a potential role of TF in chronic infection/inflammation setting. Tuberculosis is characterized by the presence of a granulomatous lesion. It is known that later stages of granuloma formation are accompanied by fibrosis which forms a barrier and have important implications in containing the spread of infection and inflammation [69]. It is thus possible that localized activation of coagulation by TF expression on the surface of activated lung macrophages in response to *Mtb* infection would lead to thrombin generation and subsequently fibrin deposition required for stable granuloma formation. In a recent study, using fibrinogen-knockout mice treated with mycobacterial trehalose dimycolate, it was shown that fibrinogen was required for proper granuloma formation [70]. In other studies, fibrinogen-deficient mice and wild-type mice treated with warfarin to suppress fibrin formation were found to display increased mortality upon peritoneal infection with *Listeria monocytogenes*, indicating the importance of fibrin in restricting the infection [71]. In agreement with these above findings, a recent study showed impaired cytokine and chemokine production, suppressed neutrophil recruitment, increased hepatic bacterial burden and mortality in fibrinogen-deficient mice following infection with *Yersinia enterocolitica* [72]. In the same study, mice with low TF activity succumbed to yersiniosis with a phenotype similar to fibrin(ogen)-deficient mice, indicating that the extrinsic coagulation pathway led to the protection.

In the absence of structurally intact granuloma formation, the bacteria may spread from the localized foci following macrophage apoptosis that results from increased bacterial burden. This phenomenon could further lead to systemic infection and inflammation through activation of endothelium either by the bacteria itself or by TF-bearing microparticles produced by activated and apoptotic macrophages [73]. Endothelial cells could actively contribute to host's innate immune response against mycobacteria. Earlier studies reported that endothelial cells are susceptible to mycobacterial infection and influence bacterial replication, dissemination and elimination [74]–[77]. These evidences along with our data showing prolonged endothelial induction of TF expression upon *Mtb* infection provides clues as to how an initial localized pulmonary infection could transform into a systemic infection. Increased TF expressing macrophages, TF-bearing microparticles and procoagulantly active endothelium could thus serve as a platform for increased thrombus formation with consequences such as DIC and DVT. However, further studies are needed in future to demonstrate the relevance of TF expression in tuberculosis disease pathology employing appropriate animal models.

## Materials and Methods

### Reagents

Gamma-irradiated H37Rv (γ-*Mtb*) whole cells and all mycobacterial components from H37Rv were obtained from ATCC through the BEI Resources under Tuberculosis Vaccine Testing and Research Materials contract (Manassas, VA). *Mtb* cell wall components, lipids and carbohydrates from BEI Resources. LPS from *Escherichia coli* 0111:B4 and polymyxin B were from Sigma (St. Louis, MO). Recombinant human factor VIIa (FVIIa) was from Novo Nordisk (Gentofte, Denmark). Purified human factor X was purchased from Enzyme Research Laboratories (South Bend, IN). Cytokines, CD14 and IFN-γ function neutralizing antibodies were from R&D systems (Minneapolis, MN). TLR2, TLR4 and TLR9 agonists were from Invivogen (San Diego, CA). TLR2 and TLR4 blocking antibodies were from Imgenex Corp. (San Diego, CA). RPMI medium and sodium pyruvate were from Invitrogen (Grand Island, NY). Human AB type serum was from Atlanta Biologicals (Lawrenceville, GA).

### Isolation and culture of monocyte-derived macrophages (MDMs) and endothelial cells

Blood (∼60 ml) was collected from healthy volunteers by venipuncture into BD Vacutainer containing sodium heparin (final heparin concentration 15 U/ml). All studies were approved by the Institutional Review Board of the University of Texas Health Science Center at Tyler. Written informed consent was provided by the study participants. Blood was diluted 1∶1 with Hank's balanced salt solution (with no CaCl_2_, MgCl_2_ or phenol red). Peripheral blood mononuclear cells (PBMCs) were isolated by differential centrifugation over Ficoll-Paque (GE Healthcare, density 1.077±0.001 g/ml). CD14^+^ monocytes were isolated with magnetic beads conjugated to anti-CD14 (Miltenyi Biotec) as described before [78]. To isolate CD14^+^ cells, first CD56^+^ cells were removed from CD14-depleted PBMCs with magnetic beads conjugated to anti-CD56 and then subjected to positive immunomagnetic selection. Since no significant differences were found between CD14^+^ monocytes isolated by immunomagnetic selection and the traditional adherence method in TF induction, for later experiments the adherence method was adopted for isolating CD14^+^ monocytes. Briefly, freshly isolated PBMCs as described above were plated in culture plates (Corning Costar) in RPMI containing 10% heat-inactivated human serum AB type and sodium pyruvate and allowed to adhere for 2 h. Non-adherent cells and loosely adhered cells were removed by aspiration and washing the adhered cells gently 2–3 times with warm RPMI serum free medium. Subsequently, adherent monocytes were allowed to mature into macrophages by culturing in RPMI complete medium for 4–5 days at 37°C in a humidified 5% CO_2_ atmosphere. Primary human umbilical vein endothelial cells (HUVECs), EBM-2 basal medium and growth supplements were purchased from Lonza (Walkersville, MD). Endothelial cells were cultured in EBM-2 basal medium supplemented with the growth supplements and 5% fetal bovine serum. Endothelial cell passages between 3 and 7 were used in the present studies.

### Bacterial strains, culture conditions and stock preparations

*Mtb* H37Rv and H37Ra were grown in 7H9 broth supplemented with 0.2% glycerol, oleic acid-albumin-dextrose-catalase (OADC) enrichment (Becton Dickinson Microbiology systems, Sparks, MD) and 0.05% Tween 80 at 37°C with continuous shaking till the absorbance at 600 nm reached 0.5–1.0. Culture suspensions were then pelleted by centrifugation at 3,750×g for 10 min and washed once with the fresh medium. Frozen stocks were made by resuspending bacterial pellet in sterile 7H9 broth containing 15% glycerol and were stored at −80°C until used. The number of bacteria in the stock was estimated by measuring absorbance at 600 nm.

### Infection of macrophages with *Mtb* H37Rv or H37Ra

MDMs cultured in 48-well or 12-well plates were either infected with *Mtb* H37Rv or H37Ra at a ratio of 1, 10 and 25∶ 1 (bacteria∶ macrophage). Infected MDMs were incubated for 2 h at 37°C in a humidified 5% CO_2_ atmosphere, and then washed twice with serum-free RPMI to remove extracellular bacteria, and the cells were continued to culture in serum containing medium for overnight. For experiments where the conditioned medium from H37Rv or H37Ra-infected macrophages was used, the infected MDMs were not subjected to washing at 2 h following the infection. The conditioned medium from the infected MDMs cultured for overnight was removed and filtered through 0.2 µm filter to remove cell debris and bacteria before it was added to fresh MDMs.

For treating MDMs with γ-irradiated *Mtb* H37Rv or various components of *Mtb* H37Rv, MDMs cultured in 48-well plates were used. Unless specified otherwise, MDMs were treated with γ-irradiated *Mtb* H37Rv (10 µg/ml) for 9 h. Experimental conditions for other treatments were described in Results or Figure legends.

### Measurement of TF procoagulant activity

Following treatments, MDMs were washed once with buffer A (10 mM Hepes, 150 mM NaCl, 4 mM KCl, 11 mM glucose, pH 7.4) and TF activity in MDMs was measured using either cell lysates or intact cells in factor X activation assay. Briefly intact cells or cell lysates were incubated with FVIIa (10 nM) in buffer B [buffer A containing 1 mg/ml bovine serum albumin, 5 mM CaCl_2_ and 1 mM MgCl_2_] for 5 min at 37°C followed by FX (175 nM). At the end of 5 to 15 min activation period, the amount of FXa generated was measured in a chromogenic assay using the substrate Chromogenix S-2765 (Diapharma) as described earlier [79].

### Measurement of TNF-α concentration

Levels of TNF-α in the supernatants of unstimulated, LPS- or γ-*Mtb* H37Rv-stimulated MDMs was determined by ELISA (eBioscience, San Diego, CA). The supernatants were collected after 6 h and 24 h of LPS- and γ-*Mtb* H37Rv treatment, respectively and centrifuged at 100×g for 10 min to remove cell debris and contaminating cells. The supernatants were then transferred to fresh tubes and stored at −70°C until TNF-α concentrations were measured by ELISA.

### Immunofluorescence confocal microscopy

MDMs grown on chambered coverglass were treated with LPS (100 ng/ml) or γ-*Mtb* H37Rv (10 µg/ml) for 6 and 9 h, respectively. The cells were then washed with PBS and fixed with 4% paraformaldehyde at 4°C for 30 min. Fixed cells, permeabilized with 0.025% Triton-X-100 for 10 min were processed for immunofluorescence staining of TF as described earlier [80]. Nuclei were stained with DAPI. The immunostained cells were viewed with an Axio ObserverZ1 microscope using 63× (oil) plan-apochromate lens. Images were acquired from a field of view at 0.4-µm Z-axis increments using LSM 510 Meta confocal system (Carl Zeiss, Germany). The laser setting wavelengths were 369±10 nm excitation and 450±30 nm emission for DAPI, 488±10 nm excitation and 525±10 nm emission for Oregon green. The images were processed using LSM Zen 2007 (Zeiss) software and imported to Adobe Photoshop (vs. 7.0) for compilation of figures.

### Real-time quantitative PCR analysis

MDMs cultured in 12-well plates after treatment with LPS (100 ng/ml) or γ-*Mtb* H37Rv (10 µg/ml) for different time periods were washed once with RPMI serum free medium and then lysed in TRIZOL reagent, 0.5 ml/well (Invitrogen, San Diego, CA). Total RNA samples were prepared and reverse transcribed to cDNA using High Capacity cDNA Reverse Transcription Kit (Applied Biosystems) following the manufacturer's instructions. TF induction was determined using a Step One Plus Real time PCR system (Applied Biosystems). PCR reaction mixtures were made of 40 ng of cDNA, 10 µM of primers, 5 µl of 2× iTaq™ SyBR® Green Supermix with ROX (Bio-Rad, Hercules, CA). Serial standard curve was made using 900 bp long TF DNA PCR product. All PCR analyses were done in triplicates. The results were normalized to endogenous HPRT. The primer sequences used were: **TF**, 5′-GGCGCTTCAGGCACTACAA-3′, 5′-TGCTTTTCCAATCTCCTGACTTAG-3′; **HPRT**, 5′ TAATTATGGACAGGACTGAACG-3′, and 5′-CACAATCAAGACATTCTTTCCAG-3′.

### Data collection and statistical analysis

All experiments were repeated atleast 3 times or more. Data shown in the figures represent mean ± SEM or a representative experiment. For calculating significance mostly unpaired t-test was used unless otherwise specifically stated.
